# Prediction of disease–gene–drug relationships following a differential network analysis

**DOI:** 10.1038/cddis.2015.393

**Published:** 2016-01-14

**Authors:** S Zickenrott, V E Angarica, B B Upadhyaya, A del Sol

**Affiliations:** 1 Computational Biology Group, Luxembourg Centre for Systems Biomedicine (LCSB), University of Luxemboug, 6, Avenue du Swing, Belvaux 4367, Luxembourg

## Abstract

Great efforts are being devoted to get a deeper understanding of disease-related dysregulations, which is central for introducing novel and more effective therapeutics in the clinics. However, most human diseases are highly multifactorial at the molecular level, involving dysregulation of multiple genes and interactions in gene regulatory networks. This issue hinders the elucidation of disease mechanism, including the identification of disease-causing genes and regulatory interactions. Most of current network-based approaches for the study of disease mechanisms do not take into account significant differences in gene regulatory network topology between healthy and disease phenotypes. Moreover, these approaches are not able to efficiently guide database search for connections between drugs, genes and diseases. We propose a differential network-based methodology for identifying candidate target genes and chemical compounds for reverting disease phenotypes. Our method relies on transcriptomics data to reconstruct gene regulatory networks corresponding to healthy and disease states separately. Further, it identifies candidate genes essential for triggering the reversion of the disease phenotype based on network stability determinants underlying differential gene expression. In addition, our method selects and ranks chemical compounds targeting these genes, which could be used as therapeutic interventions for complex diseases.

The availability of reliable methodologies for generating iPSC-derived cells^1, 2^ (induced pluripotent stem cells) has contributed to the establishment of disease modeling as a very promising approach for studying the molecular basis of disease onset and progression. Moreover, the possibility of producing patient-specific iPSC-derived cells from individuals with disease-relevant mutations offers an advantageous *in vitro* system for the study of pathogenesis and performing drug screening in differentiated human cell types.^3^ However, the multifactorial nature of many human diseases, which are characterized by the dysregulation of multiple genes and interactions in gene regulatory networks (GRNs)^4, 5, 6^ significantly hampers our understanding of molecular mechanisms related to the disease pathology. As a result, the rate at which novel drug candidates can be translated into effective therapies in the clinic is rather low.^7, 8^

In the past years, the large-scale generation of high-throughput biological data has enabled the construction of complex interaction networks that provide a new framework for gaining a systems level understanding of disease mechanisms.^9^ These network models have been useful for predicting disease-related genes based on the analysis of different topological characteristics, such as node connectivity,^1, 10^ or gene–gene interaction tendency in specific tissues.^12^ Disease-gene associations have also been predicted based on the identification of network neighbors of disease-related genes,^13, 14, 15^ or by predicting disease-related subnetworks.^16, 17, 18^ In other approaches, cellular phenotypes are represented as attractors – that is, stable steady states – in the gene expression landscape,^19^ and phenotypic transitions are modeled by identifying nodes destabilizing these attractors.^20, 21, 22^ This rationale has been used to model disease onset and progression as transitions between attractor states, in which disease perturbations, such as chemical compounds or mutations, can cause a switch from a healthy to a disease attractor state.^23, 24^

An alternative approach increasingly used explores functional connections between drugs, genes and diseases, involving the development of databases and tools integrating bioactivity of chemical compounds, chemical perturbation experiments and drug response at the cellular, tissue or organism levels.^25, 26, 27, 28^ In particular, some of these resources have been developed for connecting drugs and diseases based on gene signatures^29, 30, 31^ – for example, differentially expressed genes between disease and healthy phenotypes. For example, the Connectivity Map (CMap)^30, 31^ constitutes a widely used database of gene expression profiles from cultured human cancer cells perturbed with chemicals and genetic reagents. It has been successfully applied for predicting drug effects and mode of action in different human diseases.^32, 33, 34, 35^ However, following this approach disregards the underlying gene regulatory mechanisms. Network pharmacology strategies attempt to address this problem and identify genes whose perturbations could result in a desired therapeutic outcome.^36^ This guided rationale for drug prediction is of great importance as previous studies suggest that only ~15% of network nodes can be chemically tractable with small-molecule compounds.^37^ Moreover, molecular network robustness may often counteract drug action on single targets, thus preventing major changes at a systems level.^38^ Thus, network pharmacology methodologies are promising for the identification of optimal combinations of multiple proteins in the network whose perturbation could revert a disease state.^7, 38, 39, 40^

Nevertheless, current network and gene signature-based approaches for identifying disease-related genes and drug–disease associations have important limitations. In particular, network-based methods rely on a unique network topology while there are compelling evidences suggesting that different cellular phenotypes, such as healthy and disease states, are characterized by fairly different GRN topologies,^41, 42^ leaving these methods unable to identify differential regulatory mechanisms leading to a disease pathology. Furthermore, gene signature-based methods, such as the CMap,^30, 31^ have some important shortcomings for the selection of the right subset of genes composing a signature.^43^ Recently, new methods have been proposed to improve gene signature analysis assuming independence between the expression of different genes, leading to more reliable drug–drug^43, 44^ and drug–disease connections.^29, 45^ However, a detailed analysis of gene–gene interaction networks in a specific phenotype is generally neglected in these approaches. Moreover, to our knowledge there are no approaches combining information on perturbation of cellular phenotypes with a thorough differential network-based analysis for identifying disease–gene–drug relationships. In addition, strategies for predicting multitarget drugs are scarce and there is a lack of a robust set of design tools to routinely apply these multitarget approaches.^7^

In this paper, we introduce a novel network-based approach for predicting target genes and bioactive compounds that could revert disease phenotypes. Our method relies on the reconstruction of GRNs corresponding to disease and healthy phenotypes^21^ by compiling gene interactions from literature. Further, it performs a differential network analysis for identifying gene targets and drugs that could induce the transition from disease to healthy phenotypes. Validation of our method with perturbation data from the CMap^30, 31^ shows that in most cases our predictions of drugabble genes is in good agreement with the experimental data. Finally, our method was used to make predictions of disease-related genes and bioactive compounds in three different disease models, which have not been extensively studied. Thus, we believe that the method presented here can be useful in the identification of disease–gene–drug relationships, and therefore in guiding experimentalists in the design of effective therapeutic strategies for treating human diseases.

## Results

### Network reconstruction and differential network analysis

Network-based models of GRNs constitute an important tool for disease modeling, aiming at identifying disease-causing genes. Experimental studies suggest that the regulatory impairments leading to disease pathology are associated with different GRN topologies, underlying the phenotypic differences between healthy and disease states.^41, 42^ Thus, differential network modeling is essential for identifying differential regulatory network modules stabilizing the pathological expression pattern under study. In the approach presented here, our method infers phenotype-specific GRNs for disease and healthy states, starting from experimentally validated gene–gene interactions compiled from Thomson Reuters' MetaCore database. To obtain contextualized GRNs, our method prunes literature interaction maps compiled in each case, including interactions occurring in different tissues and organisms, and at the same time infers the mode of action of interactions having unspecified effects (see Materials and Methods for details).

We validated the inference algorithm by assessing the enrichment of validated ChIP-Seq interactions before and after contextualization (see Materials and Methods for details). Our results for these benchmarking assays show that up to 89.6% of the ChIP-seq interactions are preserved in the contextualized networks (Table 1), which demonstrate that our method is able to reconstruct fairly reliable phenotype-specific GRNs (Supplementary File S1). Moreover, we observe a high variability in the ratio of phenotype-specific and common interactions between the two networks in these examples (Table 2), indicating that both phenotypes cannot be accurately modeled by considering a single GRN topology, thus highlighting the importance of a differential network analysis approach.^41, 42^ We also performed a thorough comparison of our method with other methods available for network reconstruction of direct and signed GRNs using CellNOptR^46^ and SignetTrainer.^47^ In this comparison, we measured the enrichment in experimentally validated interactions in the reconstructed GRNs, as well as the agreement between the GRN models generated by each method and the phenotype-specific gene expression patterns in the 20 benchmarking data sets analyzed (Supplementary Table S2.1 in Supplementary File S2). The results confirm that our method generates more accurate GRNs (94% compared with 48 and 88%) while showing higher enrichment in ChIP-Seq-validated interactions (94% compared with 58 and 83%).

After obtaining healthy and disease phenotype-specific GRNs, our methodology identifies common stability determinants between these networks to derive candidate target genes, whose perturbations could revert the disease phenotype (see Materials and Methods for details). We analyzed GRN response to millions of combinations of target gene perturbations, in the following referred to as multitarget combinations, and ranked drugs according to the enrichment of their targets in these multitarget combinations (see Materials and Methods for details). At the same time, simulation assays were performed to study the effects of specific drugs on the system's attractor (see Materials and Methods for details). Combining simulation assays and enrichment analysis allow us to predict the most suitable drugs to revert the disease phenotype.

### Validation of the algorithm for inferring disease–gene–drug relationships

To show the applicability of our method for predicting genes and drugs triggering cellular phenotypic transitions, we selected six examples from the CMap,^31^ encompassing cellular gene expression changes upon chemical perturbation. These examples include the use of different chemical compounds such as celastrol+androgen, gedunin+androgen,^32^ celastrol, cobalt chloride, estradiol and genistein for treating LNCap or MCF7 cell lines. Following our methodology, we contextualized GRNs for the control and drug-induced phenotypes and assessed enrichment of the top-ranking drugs obtained from the CMap by means of simulated multitarget combinations (see Materials and Methods for details).

**Figure 1:**
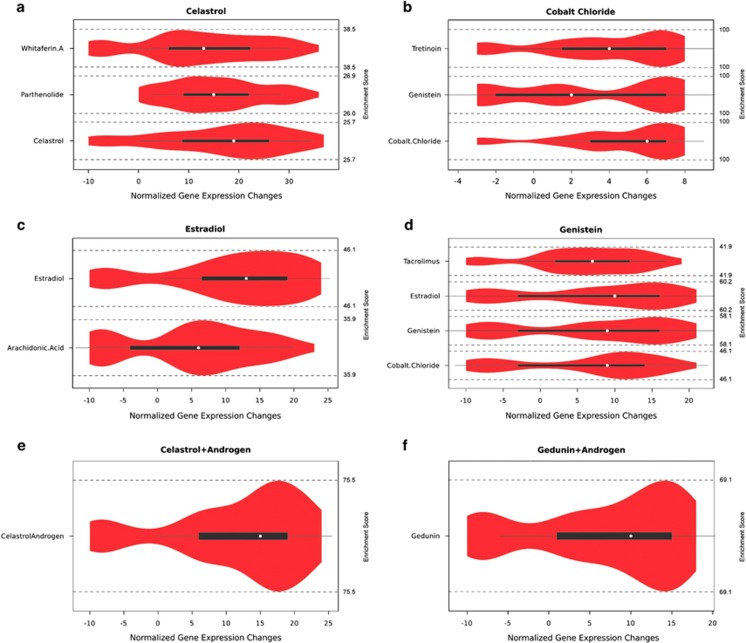
Results of drug-induced examples from the CMap. Violin plot of (**a**) celastrol, (**b**) cobalt chloride, (**c**) estradiol, (**d**) genistein, (**e**) celastrol+androgen and (**f**) gedunin+androgen showing the enrichment of drugs in the space of normalized gene expression changes resulting from simulated multitarget combinations. White dots represent the mean gene expression change of the simulated multitarget combinations underlined by the variance of the distribution (black bar). The dashed lines show the maximum enrichment score in percent for each drug in all of the cases. An optimal enrichment pattern corresponds to low scores in low normalized gene expression changes and high scores in high expression changes reflecting that the drug targets are specific to induce significant changes of the gene expression pattern. Application of cobalt chloride in (**b**) is an example of such optimal distributions

**Figure 2:**
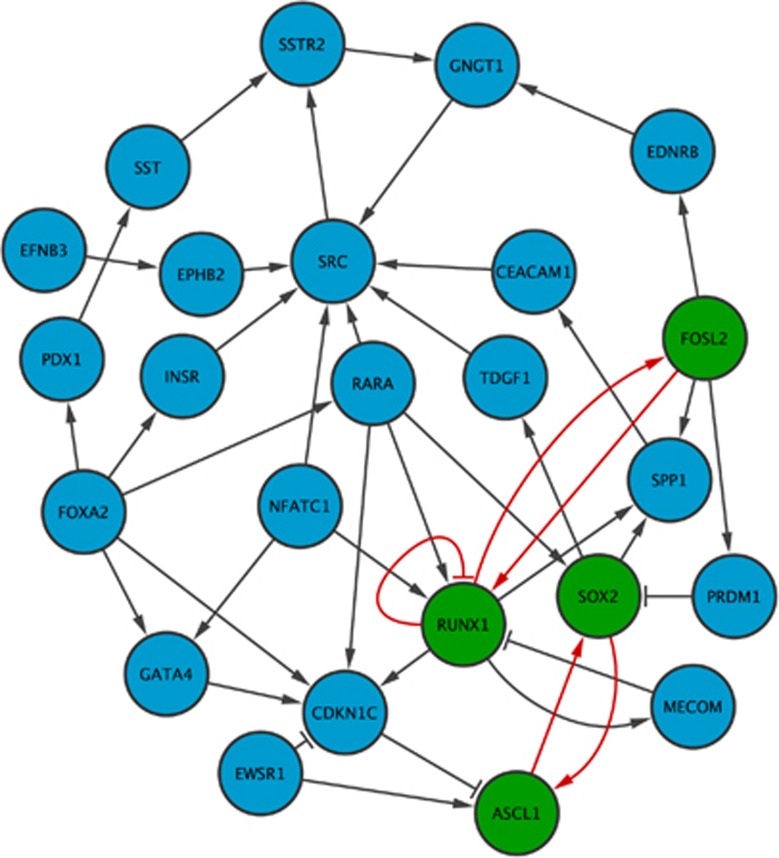
Core networks of contextualized control network. Network representation of the control network for cobalt chloride. Four genes (green) are contained in three common circuits (interactions highlighted in red), one negative autoregulation (RUNX1 —| RUNX1) and two positive feedback loops between RUNX1 and FOSL2, and ASCL1 and SOX2, respectively. No differentially regulated genes were found to be candidate genes for perturbation. The poor circuit coverage of the network is representative for the poor network response upon application of cobalt chloride. Most of the network perturbations can be recovered by the network

In all but one of the examples, we identified multitarget combinations able to revert at least 60% of the gene expression pattern (Supplementary Table S1). Furthermore, comparing the resulting enrichment distributions clearly identifies the drug inducing the phenotypical change in all cases (Table 3). Here, lower area under the curve (AUC) values correspond to better enrichment patterns, as the cumulative enrichment distribution functions are then lowly enriched in the worst multitarget combinations and highly enriched in the top-ranking ones. The deviation from the uniform AUC then provides insight into how much more specific the drugs are in comparison with an uninformative enrichment pattern (see Materials and Methods for details). Figure 1 illustrates the results of the enrichment analysis of drug targets in the simulated multitarget combinations for the drugs tested in the different examples. The normalized enrichment score corresponds to the induced gene expression changes of non-perturbed genes (see Materials and Methods). As can be seen in the examples of celastrol/gedunin+androgen, our method is also applicable to drug combinations (Figures 1e and f). Even though, in general, combinatorial effects of compounds need to be taken into account, celastrol/gedunin and androgen do not have common gene targets and thus exhibit no combined effects upon induction. Among all the examples, genistein constitutes a special case, as the AUC is lower compared with that for any other drug (Table 3), but simulation assays do not seem to confirm this as only 12.24% of the gene expression pattern is changed upon perturbation (Supplementary Table S1). In case of cobalt chloride, simulation assays of multitarget combinations containing candidate genes reveal a change of 35% of the gene expression program, upon perturbation of RUNX1 (runt-related transcription factor 1), FOSL2 (FOS-like antigen 2), ASCL1 (achaete-scute family BHLH transcription factor 1) and SOX2 (SRY (sex determining region Y)-Box 2), which belong to network stability determinants (Supplementary Table S1 and Figure 2). Topological analysis of the contextualized control network reveals only four repressive interactions, so that the repression of a gene can only be modulated by the absence of any activator, explaining the poor change in the gene expression program upon perturbation (Figure 2). However, cobalt chloride is still identified as the most suitable drug to induce the phenotypical transition (Table 3). As can be seen in Figure 1 and Table 3, in general the top-ranking solutions predicted using our methodology are enriched in genes targeted by the drugs that were used to experimentally induce the phenotypic transition.

### Predicting candidate genes and drugs from disease-control studies

We applied our methodology to disease/control case studies, including rheumatoid arthritis in B cells and systemic lupus in B and CD4+ cells for identifying differential network determinants involved in stabilizing disease and healthy phenotypes, for predicting genes that could be perturbed to revert the disease phenotypes. Further, we gathered information from the Comparative Toxicogenomics Database^48^ to identify gene–drug mode of action to propose candidates for perturbing the predicted target genes. Enrichment of the drugs was assessed by comparing the AUC of the cumulative enrichment distribution function and compared against the AUC of a uniform, uninformative enrichment distribution (see Materials and Methods for details). An overview of the common and phenotype-specific interactions for the reconstructed networks in each case can be found in Supplementary Table S5.

#### Systemic lupus (B cells)

**Figure 3:**
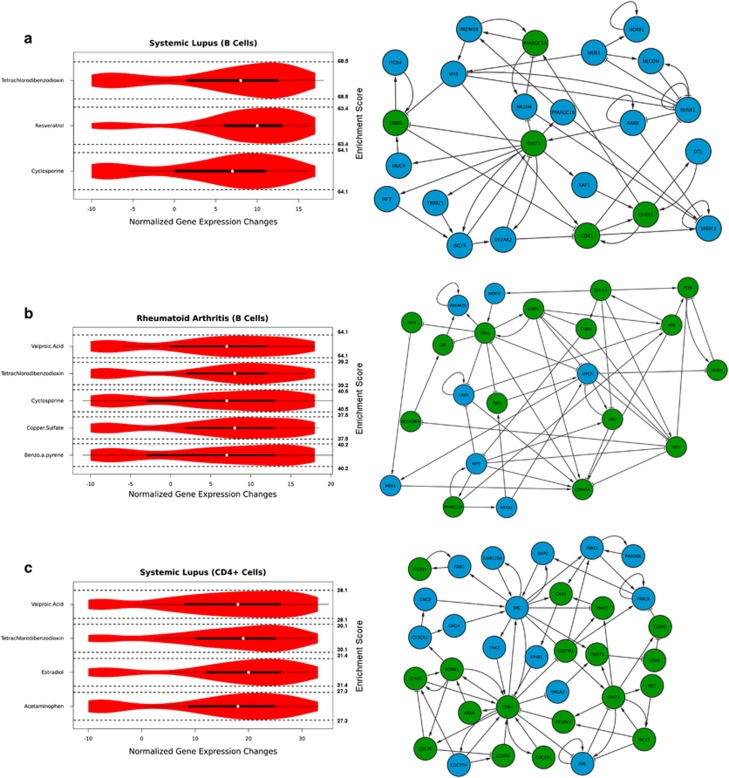
Enrichment of predicted drugs in disease-control examples. Violin plots of drug target enrichment in the space of induced normalized gene expression changes on the basis of simulated multitarget combinations. The white dots represent the mean gene expression change underlined by the variance of the distribution (black bar). The dashed lines represent the maximum enrichment (in percent) of the drugs. The enrichment patterns are contrasted with the corresponding core disease network highlighting drug targets of most enriched drug. (**a**) Enrichment patterns of drugs having the most targets in the disease network of systemic lupus in B cells reveal resveratrol to be the top-ranking drug. Its drug targets (green) are covering wide ranges of common circuits and perturb STAT1, a hub gene in the network. (**b**) Enrichment patterns of predicted drugs for rheumatoid arthritis in B cells reveal cyclosporine, copper sulfate and benzoapyrene to have similar enrichment patterns having the highest enrichment in the highest gene expression changes. Cyclosporine and copper sulfate are therefore found to be specific to significantly change the gene expression program from the disease to the healthy phenotype. The pronounced effect of copper sulfate is underlined by its targets in the core of the disease network (green) as it perturbs almost all genes. (**c**) In case of systemic lupus in CD4+ cells, estradiol and TCDD show the highest enrichments of all candidate drugs. Targets of estradiol (green) are perturbing hub genes of the network, like CDK1, and cover most of the existing circuits of the network

We used our approach for predicting drug candidates to revert the phenotype of systemic lupus in B cells (GEO: GSE4588). After reconstructing the disease and healthy specific GRNs, the differential network analysis for identifying network stability determinants rendered 11 candidate genes including STAT1 (signal transducer and activator of transcription 1), whose upregulation is associated with onset and progression of systemic lupus.^49^ The top-ranking drugs were found to be tetrachlorodibenzodioxin (TCDD) (8 targets), cyclosporine (5 targets) and resveratrol (5 targets). Notably, all identified candidate drugs perturb STAT1, indicating their prospective efficacy for treating systemic lupus. Enrichment analysis revealed that cyclosporine and TCDD share a similar enrichment pattern (Figure 3a). However, the enrichment of resveratrol shows a more pronounced effect reflected by higher scores in Figure 3a, and lower AUC of the enrichment cumulative distribution function (CDF) (Table 4). In Supplementary Table S2, we show the analysis of the network response upon drug application and the gene enrichment in multitarget combinations. The simulation assays suggest that resveratrol and TCDD induce a change in the gene expression program of 59.4% and 68.8%, respectively. Thus, application of these drugs constitutes interesting therapeutic formulations for treating systemic lupus in B cells. There exist experimental evidence supporting our predictions, as resveratrol has been found to act as an antiatherogenic agent in human macrophages.^50^ Also, a positive effect of TCDD on the pathological process of systemic lupus is suggested owing to its immunosuppressive effects in murine systemic lupus.^51^ Furthermore, cyclosporine was found to represent a helpful treatment of systemic lupus in clinical trials.^52^ Thus, our analysis reveals cyclosporine, an already known effective treatment for systemic lupus, as well as predicts resveratrol and TCDD as prospective therapeutic interventions.

#### Rheumatoid arthritis (B cells)

In this example, we predicted drug candidates for rheumatoid arthritis in B cells (GEO: GSE4588) using our methodology. We identified 27 candidate genes for perturbation, including a TCF7L2 (transcription factor 7-like 2 (T-cell specific, HMG-box)) polymorphism assumed to be associated to rheumatic arthritis^53^ and CDKN1A (cyclin-dependent kinase inhibitor 1A (P21, Cip1)), whose decreased expression has been linked to an increased risk to develop autoimmune diseases, such as rheumatoid arthritis.^54^ The drugs having more gene targets in the disease network are benzo(*a*)pyrene (14 targets), copper sulfate (12 targets), TCDD (12 targets), valproic acid (12 targets) and cyclosporine (10 targets). Simulation assays of multitarget combinations show that both TCF7L2 and CDKN1A are included in all combinations inducing the most significant phenotypic change. Enrichment analysis reveals copper sulfate to have the highest enrichment in the top-ranking multitarget combinations, followed by benzo(*a*)pyrene, TCDD and cyclosporine (Figure 3b and Table 3) underlined by the lower AUC of copper sulfate showing its more specific enrichment in the best multitarget combinations. Simulation assays reveal that copper sulfate reverts 53% of the disease phenotype, whereas cyclosporine only reverts 34% (Supplementary Table S3). Indeed, most of the genes in the core of the disease network are targeted by copper sulfate, explaining its pronounced effect on the gene expression program. In contrast, application of benzo(*a*)pyrene results in an unstable behavior of the network and is therefore not be considered to cause the phenotypic transition in this disease. Previous studies support these conclusions, showing the beneficial effects of cyclosporine in the short-term treatment of rheumatoid arthritis,^55^ whereas copper sulfate constitutes a novel approach whose effect on this disease needs to be further elucidated.^56^

#### Systemic lupus (CD4 cells)

As a last example, we predicted candidate genes and drugs for treating systemic lupus in CD4 cells, starting from the same gene expression data set used in the first example (GEO: GSE4588). We identified 35 candidate genes for perturbation, including STAT1 and other genes relevant to disease pathology, such as IRF7 (interferon regulatory factor 7)^57^ and ISG15 (ISG15 ubiquitin-like modifier).^58^ We identified four drugs targeting the predicted candidate genes, including TCDD(15 targets), acetaminophen (12 targets), estradiol (15 targets) and valproic acid (11 targets). The enrichment analysis of multitarget drug combinations shows that estradiol and TCDD are highly enriched in the predicted candidate genes (Figure 3c), which is further underlined by the AUC value as a deviation of >20% from the uniform AUC proves its higher enrichment in the top-ranking multitarget combinations (Table 4). Simulation assays show that TCDD and estradiol are able to revert 72% and 62% of the disease gene expression program, respectively (Supplementary Table S4), which makes them suitable drugs to treat this disease. As stated previously, TCDD acts as an immunosuppressor in murine systemic lupus,^51^ and recent experimental results show the protective effects of estradiol against lupus-mediated hypertension and proteinuria in adult female mice,^59^ giving support to our predictions in this case.

## Discussion

In this work, we propose a method for predicting disease–gene–drug relationships based on the reconstruction of phenotype-specific GRNs underlying phenotypic differences between disease and healthy states, solely relying on differential gene expression data. By following this rationale, we are able to generate more realistic network models to study disease mechanisms by analyzing differences in gene regulatory interactions underlying different cellular states.^41, 42^ We benchmarked our method for assessing its reliability for differential network inference and showed that our method generates fairly reliable context-specific networks, which are highly enriched in ChIP-Seq validated interactions contained in ENCODE^60^ (see Supplementary Table S1.2). We also performed a thorough comparison of our method with two available methods for modeling direct and signed GRNs, namely CellNOptR^46^ and SignetTrainer.^47^ The results from this comparison (Supplementary Table S2.1 in Supplementary File S2) demonstrate that the GRN models built with our method are more reliable and enriched in experimentally validated interactions than the models generated by other similar methods. After reconstructing phenotype-specific GRNs, we performed a differential network analysis for identifying network motifs determining the stability of disease and healthy phenotypes. In this regard, combinations of positive and negative circuits have been shown to have an important role in the stability of GRNs.^21, 61^ To derive candidate genes for perturbation, our method first selects optimal combinations of genes belonging to these common circuits between healthy and disease GRNs. Perturbation of these genes should be able to trigger the disease to healthy state phenotypic transition. Second, our method also selects genes under differential regulation – that is, genes regulated by different sets of transcription factors – not belonging to common circuits, as they can only be perturbed individually.

We validated the performance of our methodology for inferring disease–drug relationships by analyzing six data sets of known drug-induced phenotypical changes obtained from the CMap.^31^ Our results show that by using our differential network approach, we are able to accurately predict genes and drugs associated with the phenotypic changes, as in most cases the top-ranking solutions are highly enriched in drug gene targets causing the reversion of the disease phenotype *in vitro.*^31^ In all validation examples, perturbations of candidate genes in the GRN models cause a change in the global gene expression between healthy and disease states ranging from 42 to 80%. The only exception is the genistein case, in which the number of genes in the reconstructed network model targeted by chemical compounds in databases is rather low.^48^ These results highlight that differential topological analyses of GRN models recapitulate phenotypic differences between healthy and disease states, and can be successfully used for identifying genes whose perturbations can induce disease reversion. Thus, unlike current approaches relying on single network topologies, our method is based on a more realistic framework that allows us to more accurately predict genes whose perturbations have a desired phenotypic change, and therefore improving strategies in network pharmacology.^37^ In addition, we applied our methodology to three disease-control case studies, for which there is differential gene expression data, to predict disease–gene–drug relationships constituting prospective clinical treatments being subject to future validations.

Previous approaches aiming at identifying drug perturbations for reverting the disease phenotype solely relied on gene signatures inferred from differential gene expression,^29, 45^ or regulatory modules targeted by drugs,^62, 63^ without considering gene interactions underlying disease and healthy states. However, the regulatory mechanisms underlying disease pathologies could provide important mechanistic insights on cellular responses to drug application,^64^ and interactions among genes targeted by the drugs in GRNs. Thus, our proposed differential network analysis approach overcomes this drawback by *in silico* simulating network response upon drug application of genes in GRN stability determinants. Indeed, drugs predicted with our approach for reverting the disease-related expression patterns have been validated in experimental studies. In all cases, the three candidate drugs identified to have the most pronounced effects on reverting disease gene expression programs have been used for treating the pathological process of systemic lupus and rheumatoid arthritis. In particular, cyclosporine has been successfully applied in the process of treating systemic lupus and rheumatoid arthritis.^52, 55^ However, we predicted resveratrol as a novel treatment of systemic lupus, whose potential therapeutic utility is underlined by previous studies.^50^ Based on these evidences, we are not only able to predict suitable drugs to revert disease-related gene expression patterns but also to significantly narrow down the set of candidate drugs by means of a differential network analysis.

The differential network-based approach to identify disease–gene–drug relationships has been validated with predictions in drug-induced phenotypes in six examples. Interestingly, in all the studied examples we predicted multitarget drug combinations inducing more efficient transitions between disease and healthy phenotypes *in silico*. This could constitute a promising application of our network-based approach for predicting multitarget drug formulations for treating human diseases. Moreover, the application of our approach to systemic lupus and rheumatoid arthritis identified well-known drugs already used in current clinical therapies as well as new prospective treatments subject to future clinical trials. Thus, our method offers a helpful tool to identify disease–gene–drug relationships of other pathological processes to guide experimentalists in the discovery of more effective treatments.

## Materials and Methods

### Compilation of gene–gene interaction maps from literature resources

To obtain the initial gene interaction maps used throughout this study, we first identified the differentially expressed genes between two cellular phenotypes using an independent two-sample *t*-test. In these interaction data sets, we only take into account those genes having a *P*-value lower than and a fold change higher than the defined case-specific cutoffs that are set depending on the number of genes profiled in each case, and the number of replicates for performing the statistical analysis. After defining the sets of differentially expressed genes in each case, we reconstructed gene interaction maps from experimentally validated interactions reported in scientific publications. For this purpose, we use the information contained in MetaCore from Thomson Reuters, a highly curated resource of biomolecular interactions compiled from literature, to retrieve all direct interactions among differentially expressed genes. Notably, interactions whose effect – that is, activation or inhibition – is unknown are also included in the interaction maps for inferring their corresponding mode of action during the following steps of our methodology. Even though we use validated interactions reported from literature throughout this paper, our methodology is designed to be general and can take advantage of interaction maps generated using different approaches, such as gene coexpression analysis.

### Reconstruction of phenotype-specific GRNs

The interaction maps compiled from the literature are rather noisy, comprising experimentally validated interactions reported in different cell lines, tissues or organisms, which make it difficult to identify the gene interactions taking place in the specific phenotype under study. As the attractors of the initial network are not necessarily matching the studied biological conditions – that is, transcriptomic data – it is necessary to perform a contextualization of the literature interaction maps to derive the phenotype-specific GRNs, as previously described in other recent reports.^21, 65^ To accomplish this task, our method implements a genetic algorithm (GA) to contextualize two different GRNs, and makes them compatible to the gene expression profiles of two different cellular phenotypes, such as disease and healthy states. Here, a Boolean modeling formalism with synchronous updating scheme^66^ is used to represent the GRNs to capture the steady state behavior of a molecular network.^21^ Network dynamics are captured by the evolution of each node at the same time, defined by an updating rule that depends on the node's incoming regulatory interactions. Throughout this study, we assume a ‘majority rule' – that is, a gene is expressed (ON) if it has more incoming activating interactions than incoming inhibiting interactions, and not expressed (OFF) otherwise. Following this logic, a gene having as many activators as inhibitors is considered to be not expressed. However, a gene that is not regulated by any other gene constitutes an exception from this rule as it is assumed to be expressed.

**Figure 4:**
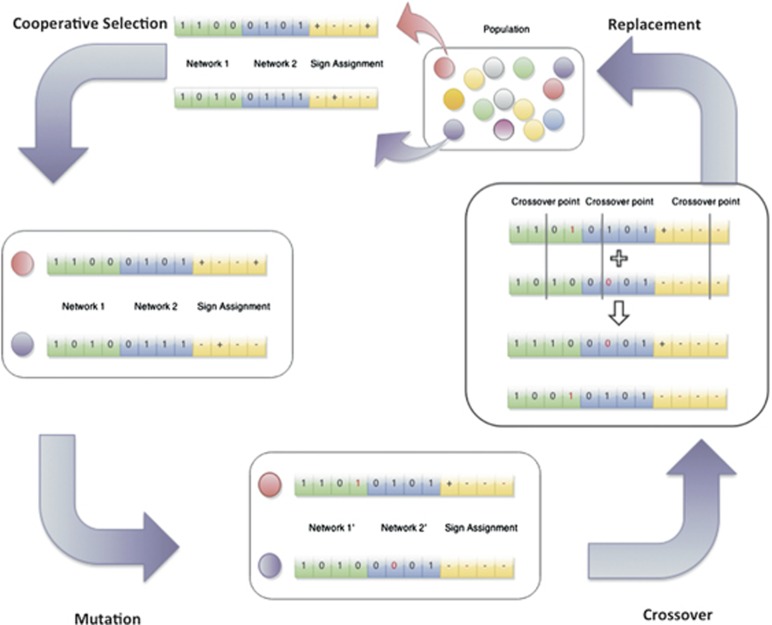
Workflow of the GA. Starting from a randomly generated population of binary representations of two phenotype-specific networks and the mode of action corresponding to the contained interactions, the GA iteratively selects two individuals, mutates them – that is, prunes or restores some of their interactions – and recombines them. Each individual contains interaction information of the control network (green), the disease network (blue) and a mode of action assignment (yellow). The mode of action is either activation (+) or repression (−). Finally, the two newly derived individuals replace the worst individuals in the population. Based on simulation assays, the agreement between network attractors and transcriptomic data is assessed for each individual. A score is derived in taking into account network attractors and stability

As previously mentioned, the initial interaction maps may contain interactions in which the effect of the interaction (activation or inhibition) among genes is unknown – that is, unsigned interactions. During contextualization, a sign for these interactions is inferred that is consistent with both phenotype-specific networks. The GA is comprised of a population of individuals implemented using a binary array containing an interaction map for both phenotype-specific networks and an inferred sign assignment (Figure 4). The presence of an interaction in a network is represented following a Boolean encoding, letting ‘1' represent its presence and ‘0' its absence. In the same manner, we encode the signs of interactions, letting ‘1' represent activation and ‘0' inhibition. Consequently, the array representing one individual is split into three subarrays (Figure 4). The first subarray (green) represents the network corresponding to the first phenotype (healthy), and the second subarray (blue) represents the network of the second phenotype (disease), whereas the last subarray (yellow) corresponds to the inferred signs. As we start from the same initial interaction map to contextualize against both phenotypes, the inferred signs have to be identical in both networks.

The complete workflow of the network inference algorithm can be described as follows: for each phenotype-specific GRN, the GA generates a new population of individuals – that is, a set of candidate solutions – in each generation, resulting from the following three-step approach (Figure 4):

(1) *Selection*: The selection step selects two individuals from the whole population using our own implementation of cooperative selection^67^ to preserve diversity in the population while ensuring the selection of the best individuals at least once. Following this strategy, the best individual is selected and its fitness value is updated to the average of the second and third best fitness values. Thus, this procedure ensures the selection of a different individual in the next selection step – the second best individual – preserving diversity among the best solutions while still disregarding the worst solutions in the population.

(2) *Mutation*: The second step probabilistically alters the selected individuals, by pruning or reintroducing interactions of the network. Moreover, inferred signs are also subject to probabilistic mutations resulting in a flip from activation to inhibition or *vice versa*. The choice of the mutation probability is, like the choice of the selection scheme, crucial for the performance of the GA. A high mutation probability leads to a more representative exploration of the search space. On the other hand, a low mutation probability allows the GA to converge faster towards an attractor state, even though it may be only a local optimum. The mutation parameter is set to 0.01 per subarray for all examples in the study – that is, 1% of the bits are mutated in every subarray. This conservative choice of the mutation probability allows the algorithm to converge faster while a higher exploration of the search space can still be obtained by increasing the user-defined size of the population.

(3) *Crossover*: The third step is the probabilistic recombination of the selected individuals. With a given probability *p*, which is set to 0.9 for all the examples discussed in this paper, the two individuals are recombined and, consequently, remain unchanged with probability *1−p*. As we are analyzing individuals representing two different network topologies, we adapted the traditional single-point crossover to a three-point crossover method. To account for the distinct subarrays for each individual, we choose one crossover point for the first network, another crossover point for the second network and a third crossover point for the sign assignment. After recombination we obtain two new individuals, the first one of which contains the first interaction subarray of the first individual and the second subarray of the second individual, whereas the other one contains the first subarray of the second and the second subarray of the first individual (Figure 4). We choose this crossover operation to ensure that each phenotype-specific network is treated independently during the pruning and sign estimation process.

The three aforementioned steps are implemented within the jMetal^68^ implementation of NSGA-II,^69^ an elitist multiobjective GA. The number of generations of the three steps characterizes the termination criterion of the GA. We set the population size to 700, thus being at least almost five times higher compared with the number of interactions, and the number of generations to 250 for each example. In general, these parameters have been shown to provide the best solutions. For a detailed assessment of the parameters see Supplementary File S4. After the user-defined number of iterations is exceeded, the algorithm outputs the latest population. Even though we observed that in most cases the trend of the population usually converges, it is very likely to obtain some level of variability in the solutions. Therefore, our method takes the top-ranking solutions and creates a consensus solution to narrow the different solutions down to the most likely GRN representation. Similarly, in the contextualization process described above, during this step both cellular phenotypes are treated independently.

### Inference of the effect of unsigned interactions

The estimation of the effects of interactions for which there is no experimental evidence in MetaCore is performed dynamically during the contextualization step. Instead of considering only local network properties, such as gene expression of a regulated gene and its regulator, in our method sign prediction takes into account global network properties. Based on network stability – that is, the convergence to a steady state – and the correspondence between the steady state of the GRN and transcriptomic data, a consistent interaction sign assignment is inferred. Note that the sign inference procedure allows more freedom to the GA to identify network topologies best matching the transcriptomic data. During the analysis of unsigned interactions, network consistency with gene expression data can be increased by pruning network interactions, and also by inferring the best combinations of interaction effect throughout the network. As both GRNs are reconstructed from the same initial interaction map, the inference of interaction effect is performed simultaneously for the two context-specific network representations. We assessed our method's ability to not only infer consistent assignments but also biologically validated ones in Supplementary File S3.

### Validation of the network inference algorithm

We benchmarked the performance of our algorithm for reconstructing GRNs by building six networks based on the pairwise differential expression of four well-characterized genome-wide gene expression experiments annotated in ENCODE.^60^ In particular, these experiments were performed in three cancer cell lines (GM12878, K562 and HepG2) as well as one embryonic stem cell line (H1-hESC). For each of the six differential expression patterns, starting from a literature-derived interaction map using MetaCore, we assessed the enrichment of ChIP-seq experimentally validated interactions before and after contextualization, to evaluate the performance of our method for identifying phenotype-specific interactions. We also performed a benchmarking of our methodology for reconstructing phenotype-specific GRNs, in comparison with other methods available. We gathered 20 interaction data sets, in which the great majority of the interactions have been experimentally validated (ChIP-seq interaction data from ENCODE^60^), and analyzed them with our method, CellNOptR^46^ and SignetTrainer^47^ for assessing the accuracy of the reconstructed GRNs for explaining the phenotype-specific gene expression patterns, and the enrichment in experimentally validated interactions in the reconstructed GRNs (Supplementary Table S2.1 in Supplementary File S2).

### Selection of validation examples for predicting drugs

We selected six examples for the validation of our methodology from the CMap using different small compounds including celastrol+androgen, gedunin+androgen,^32^ celastrol, cobalt chloride, estradiol and genistein for treating LNCap or MCF7 cell lines. The examples of celastrol/gedunin+androgen were chosen to illustrate that our method is able to cope with combinations of applied drugs while the others are selected from all available data sets in the CMap having at least three replicates for the control and drug-induced phenotype, and distinct pharmacological mechanisms. Estradiol targets the estrogen receptor and genistein belongs to the class of flavones and is an agonist of the estrogen receptor. Celastrol is a quinone methide triterpene inhibiting directly kinases IKK*α* and IKK*β* and inactivates CDC37 (cell division cycle 37) and p23 (prostaglandin E synthase 3) proteins and is thus an HSP90 (heat-shock protein 90 kDa) inhibitor.^70^ In this context, gedunin has been found to have similar effect and acts as an HSP90 inhibitor as well.^32^ Ultimately, cobalt chloride is lowering NF-*κ*B (nuclear factor of kappa light polypeptide gene enhancer in B cells) DNA-binding activity and results in higher expression levels of IL-6 (interleukin-6) and TGF*β* (transforming growth factor-*β*) in the brain.^71^

### Selection of candidate genes for network perturbation

We implemented the Johnson's algorithm^72^ to detect all elementary network circuits in both reconstructed GRNs, including self-loops. An elementary circuit is a path starting from and ending in the same node visiting each intermediate node only once. We then intersect the set of elementary circuits obtained for both phenotype-specific networks, including positive and negative circuits – that is, between circuits with an even number of inhibitions (positive circuits) and an odd number of inhibitions (negative circuits). Positive circuits have been shown to have an important role in maintaining network stability.^73, 74, 75, 76, 77^ On the other hand, negative circuits are a necessary condition for a network to have an attractive cycle.^78^ However, it has been shown that combinations of positive and negative circuits are able to maintain a stable steady network state.^61^ Perturbations of positive circuits have been used in previous studies to induce a transition between two different phenotypes modeled using a single network topology for describing the two phenotype-specific attractor states.^21^ In this representation, any of the genes contained in a circuit is assumed to be a perturbation candidate and a minimal combination is derived. Nevertheless, our approach takes into consideration the differences between the underlying GRN determining cellular phenotypes, and predicts as candidate genes for perturbation of all the genes contained in positive and negative circuits determining network stability. Owing to the topologic differences between phenotype-specific GRNs, network circuits do not cover similar parts of the networks, and it is more difficult to identify the genes triggering a transition between the two cellular states. Hence, our method relies on a differential network topology analysis to identify genes under differential regulation – that is, genes regulated by different sets of transcription factors – as they constitute ideal perturbation targets, which can only be perturbed individually. The sets of regulators are constrained to expressed genes, as genes that are not expressed cannot contribute to the expression of their targets in the network. The accordingly identified genes are not necessarily those genes responsible for disease onset but are rather genes able to revert most of the gene expression program upon perturbation, which makes them good candidates for pharmacological targeting.

### Identification of drugs and multitarget combinations for reversing the disease phenotype

Once the set of candidate genes for perturbation are identified, we simulate multitarget combinations of up to ten candidate genes. Usually, we are interested in a minimal multitarget combination leading to a significant change in the gene expression program between the two cellular phenotypes, which was set to a change of at least 50% of the genes in the GRNs. The size of a multitarget combination is defined as the number of genes contained in it. For each multitarget combination size between 1 and 10 we simulate network response of up to one million combinations and rank the results according to the number of genes whose gene expression is reversed between the two phenotypes. The results are normalized by substracting the number of perturbed genes from the number of genes whose gene expression is changed upon perturbation. We refer to this normalized gene expression changes in this paper as the multitarget combination score. For relating the determined candidate genes to drugs, we use two different approaches. The first approach, which is used during the validation of our methodology, solely relies on the differential gene expression pattern between the two conditions under study. The differential expression is used as an input for the CMap^31^ to obtain drugs showing a similar differential expression pattern. We select the top-ranking drugs and determine their target genes by using the Comparative Toxicogenomics Database.^48^ Later, we compute the enrichment of drug targets in the simulated multitarget combinations. In this approach, each gene is assigned a different weight according to its simulated single perturbation effect, and we analyze the enrichment of the gene targets for the compounds causing the phenotypic transition according to the CMap,^31^ in the top-ranking multitarget combinations obtained with our method. In addition, network response to induction of a single drug is measured and the drugs are ranked in accordance to the enrichment and this simulation assay analysis. In these simulation assays, the effect of chemical compounds is assumed to be constant during the trial, and the expression values of perturbed genes are kept constant throughout the whole simulation – that is, the drugs are assumed to have dominant effect on the target genes. The second approach solely relies on the Comparative Toxicogenomics Database^48^ and is used for the prediction of disease–gene–drug relationships in systemic lupus and rheumatoid arthritis. Our method predicts drugs that could have an effect on triggering the phenotypic transition by first identifying the genes in network stability determinants as described above. Then, we identified gene–drug associations by compiling drug mode of action from the Comparative Toxicogenomics Database.^48^ We then select those drugs having more gene targets among the candidate genes and perform enrichment and simulation assay analysis as described in the previous case.

### Analysis of drug–gene target enrichment

To score the drugs obtained from our differential network analysis, we compute the enrichment of drug–gene targets in the set of simulated multitarget combinations. Owing to incomplete information about drug–gene mode of action, not all drug gene targets are represented in our network. Hence, the predicted candidate genes, and therefore the multitarget combinations, do not contain all the drug–gene targets in most cases. Our analysis is therefore restricted to target genes already predicted as perturbation candidates. To study the extent of phenotypic change upon GRN drug perturbation, a weight *w_g_* is assigned to each perturbation candidate, corresponding to the normalized gene expression changes resulting from single-target perturbation of that gene. Then, for each simulated multitarget combination *i*, an enrichment score 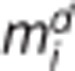 is derived by summing the weights 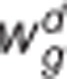 of targets of drug *d* contained in the multitarget combination – that is, 
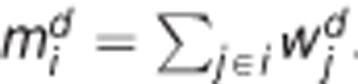
. To obtain values between 0 and 1, these scores are normalized to obtain 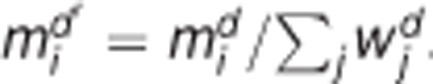. From the values obtained for each multitarget combination, a distribution is drawn over the space of normalized gene expression changes. It is determined by the probability mass function 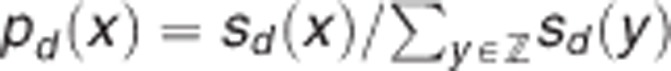, where 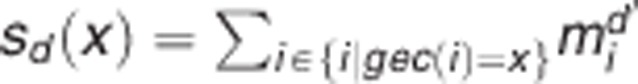 and *gec*(*i*) is the normalized gene expression change of multitarget combination *i*. To assess finally the quality of a drug with respect to its effect on the phenotype, we derive the cumulative distribution function (CDF) of the scores for each drug and calculate the AUC. Hence, the CDF of enrichment of drug *d* can be formulated as the step function 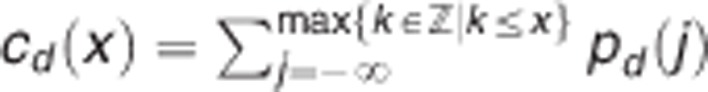 and the area under the curve is determined as 
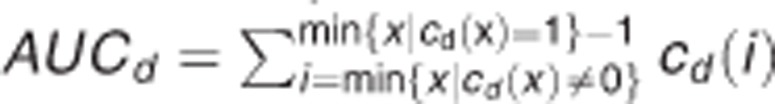
. Following the definition of *c*_*d*_ as a monotonously increasing function, an optimal enrichment pattern is reflected by *AUC*_*d*_=0. Furthermore, smaller values of *AUC*_*d*_ reflect a more favorable drug enrichment pattern as they indicate a lower enrichment in the worst multitarget combinations and at the same time a higher enrichment in the high-ranking combinations.

## Figures and Tables

**Table 1:** ChIP-Seq validation of phenotype-specific network inference algorithm

	**HepG2/GM**		**HepG2/H1**		**HepG2/K562**		**GM/H1**		**GM/K562**		**H1/K562**	
	**HepG2**	**GM**	**HepG2**	**H1**	**HepG2**	**K562**	**GM**	**H1**	**GM**	**K562**	**H1**	**K562**
Raw	92	36	122	20	74	0	2	0	88	18	4	4
Pruned	86	30	111	13	71	0	2	0	79	13	4	3
Retained (in %)	93.5	83.3	91	65	95.9	—	100	—	89.8	72.2	100	75

For six different examples, we compared the number of initially contained interactions from ChIP-Seq (Raw) to those contained in the phenotype-specific networks. On average the network inference algorithm retains 86.6% of ChIP-Seq interactions in the contextualized networks.

**Table 2:** Statistics of validation networks for the network inference algorithm

	**HepG2/Gm12878**	**HepG2/H1-hESC**	**HepG2/K562**	**Gm12878/H1-hESC**	**Gm12878/K562**	**H1-hESC/K562**
Common	424	608	430	298	662	594
Phenotype1 total	530	824	520	324	999	859
Phenotype1 specific	106	216	90	26	337	265
Phenotype1 ratio	20.00%	26.20%	17.30%	8.00%	33.70%	30.80%
Phenotype2 total	508	707	498	323	819	720
Phenotype2 specific	84	99	68	25	157	126
Phenotypes2 ratio	16.50%	14.00%	13.70%	8.30%	19.20%	17.50%

For six different examples, the number of common and phenotype-specific interactions is dissected. The percentage of phenotype-specific interactions varies markedly between 8.0 and 33.7%, highlighting the necessity of a differential network approach to accurately represent two different phenotypes.

**Table 3:** Statistical comparison of simulated drugs in the validation examples

**Case**	**Drug**	**AUC**	**Difference from AUC of uniform distribution**
Celastrol+ androgen	**Celastrol+ androgen**	**12.645**	**25.61%**
Gedunin+ androgen	**Gedunin+ androgen**	**10.423**	**25.55%**
Estradiol	Arachidonic acid	18.683	−0.99%
	**Estradiol**	**12.541**	**26.22%**
Genistein	Cobalt chloride	14.628	5.62%
	Estradiol	13.772	11.15%
	**Genistein**	**12.855**	**17.06%**
	Tacrolimus	14.532	0.62%
Cobalt chloride	**Cobalt chloride**	**4**	**27.27%**
	Genistein	4.833	12.12%
	Tretinoin	4.134	24.83%
Celastrol	**Celastrol**	**19.776**	**15.85%**
	Parthenolide	21.943	0.66%
	Whitaferin A	24.471	−0.41%

In the six drug-induced examples taken from the CMap, the area under the curve (AUC) of the cumulative enrichment distribution function is shown and compared against the AUC of a uniform distribution. Lower AUC values and higher difference from the uniform distribution indicate an enrichment of drug targets in multitarget combinations able to induce a higher change of the gene expression program. The most enriched drugs are highlighted in each case (bold) and agree with the experimentally induced drug.

**Table 4:** Statistical comparison of simulated drugs in systemic lupus and rheumatoid arthritis

**Case**	**Drug**	**AUC**	**Difference from AUC of uniform distribution**
Systemic lupus (B cells)	Cyclosporine	11.722	13.17%
	**Resveratrol**	**8.918**	**33.94%**
	Tetrachlorodibenzodioxin	11.61	14.00%
Systemic lupus (CD4 cells)	Acetaminophen	18.721	12.93%
	**Estradiol**	**16.627**	**22.66%**
	Tetrachlorodibenzodioxin	17.111	20.41%
	Valproic acid	18.615	13.42%
Rheumatoid arthritis (B cells)	Benzo(*a*)pyrene	11.892	15.05%
	**Copper sulfate**	**11.578**	**17.30%**
	Cyclosporine	12.169	13.08%
	Tetrachlorodibenzodioxin	12.084	13.69%
	Valproic acid	12.695	9.32%

For systemic lupus in B and CD4 cells and rheumatoid arthritis in B cells, the area under the curve (AUC) of the cumulative enrichment distribution function is shown and compared against the AUC of a uniform distribution. Lower AUC values and higher difference from the uniform distribution indicate an enrichment of drug targets in multitarget combinations able to induce a higher change of the gene expression program. The most enriched drugs are highlighted in each case (bold) and agree with the experimentally induced drug.

## Abbreviations

iPSC: induced pluripotent stem cell
CMap: Connectivity Map
GRN: gene regulatory network
AUC: area under the curve
CDF: cumulative distribution function
GA: genetic algorithm
TCDD: tetrachlorodibenzodioxin
RUNX1: runt-related transcription factor 1
FOSL2: FOS-like antigen 2
ASCL1: achaete-scute family BHLH transcription factor 1
SOX2: SRY (sex determining region Y)-Box 2
STAT1: signal transducer and activator of transcription 1
TCF7L2: transcription factor 7-like 2 (T-cell specific, HMG-box)
CDKN1A: cyclin-dependent kinase inhibitor 1A (P21, Cip1)
HSP90: heat-shock protein 90 kDa
