# Supplementary material for: Prediction of disease–gene–drug relationships following a differential network analysis
Source: Cell Death Dis. 2016 Jan 14;7(1):e2040–. doi: 10.1038/cddis.2015.393 (PMC4816176; doi:10.1038/cddis.2015.393)
Supplement: Supplementary Material S1 [file cddis2015393x1.docx]

Supplementary Material

**Validation with phenotype-specific ChIP-Seq data**

In order to validate our method, we reconstructed networks corresponding to six different genome-wide gene expression profiles annotated in ENCODE. We gathered expression data for B-lymphocytes (GM12878), embryonic stem cells (H1-hESC), leukemia related lymphoblasts (K-562) and cells from a differentiated hepatocellular carcinoma (HepG2) from Duke Affy Exon experiments. Then, we pairwise combined them to form six different examples of differential networks associated to different phenotypes. For each pair, we obtained a list of differentially expressed genes by conducting a t-test and setting a threshold for both p-value and fold-change. Unless stated otherwise, we choose a p-value less than 0.001 and a fold-change greater than 4. Next, the list of differentially expressed genes was used to obtain an initial literature interaction map from MetaCore from Thomson Reuters, each one comprising both signed and unsigned interactions. To validate our methodology, we gathered ChIP-Seq data from ENCODE for all aforementioned cell lines (see Table S1.1) to compare the interactions included in the reconstructed networks with experimental TF-DNA interaction. Therefore, we compare the amount of interactions reported in ChIP-Seq before and after network contextualization, which shows that non-relevant interactions are correctly pruned out. Table S1.2 sums up the results for all examples and shows that most interactions reported in ChIP-Seq are accordingly included in the contextualized networks. In addition, our results also show that all pruned interactions are pruned due to either maintaining network stability or because of inconsistencies with expression data. Figure S1.1 illustrates the core of the contextualized networks for six examples highlighting common interactions in green and phenotype-specific interactions in black. Since the ratio of common and phenotype-specific interactions (see Table 2) differs significantly from in each case, the necessity of differential network analysis is further underlined. The phenotype-specific networks derived for Gm12878 and H1-hESC, for example, are highly compatible whereas the networks of Gm12878 and K-562 are significantly different with respect to the ratio of network-specific interactions. This diversity in network-specific interactions underscores the necessity of a differential network approach rather than explaining two phenotypes within a single topology.

***HepG2/GM12878***

In this benchmarking test we studied the phenotype specific networks for HepG2 and GM12878. For a p-value of 0.001 and a fold change greater than 4, we obtained a list of 775 differentially expressed genes. The compiled initial interaction map comprises 344 out of the 775 genes, forming 665 interactions among each other. We obtained ChIP-Seq data for 9 and 15 TFs from ENCODE for HepG2 and GM12878, respectively, comprising 92 interactions for HepG2 and 36 for GM12878.

Following our network reconstruction approach, the HepG2 specific contextualized network contains 86 out of 92 interactions. Among the six pruned interactions the interaction between *CEBPB* and *RAC2* is pruned because *CEBPB* is considered to be up-regulated whereas *RAC2* is down-regulated. Due to the network topology, this interaction could contribute to network stability if it is predicted to be inhibition. But, due to the consensus between the best solutions generated by our method, it is not considered. The inhibition of *GPAM* by *FOXA1* is pruned correctly, because both genes are up-regulated. As *CEBPB* is acting as an activator on *BCL2A1*, this interaction is inconsistent with gene expression. In the interaction between *CEBPB* on *BCL2A1, CEBPB* is up-regulated whereas *BCL2A1* is down-regulated and as the latter is only regulated by the former, this interaction should be pruned. Similarly to the first example, the interaction between *HNF4A* and *C2* does not contribute to neither gene expression explanation nor network stability. Both *C2* and *HNF4A* are up-regulated and *C2* has another activator making this interaction redundant, even though it can be clearly identified as an activation. Following the same rationale, the edge between *HNF4A* and *SERPINC1* is pruned accordingly. The last interaction under consideration is the activation of *CCL22* by *CEBPB*. Here, *CEBPB* is the only up-regulated gene acting on *CCL22*, whereas its expression value is down-regulated. Thus, pruning this interaction is the only possible explanation for the gene expression patterns observed for those genes.

The GM specific network lacks also six interactions present in the ChIP-seq network. In this case all pruned inhibitory interactions are due to the existence of incompatibilities with the gene expression profile. In case of *STAT5A* acting on *IRF8* and *PAX5* acting on *PRDM1* the interaction is pruned because all genes are considered to be up-regulated. In case of *RUNX3* inhibiting *NTRK2*, both genes are down-regulated resulting in no effect of these interactions in the network. Similarly, the other three interactions (inhibitions of *RHOB* by *MEF2C*, *FCER2* by *CEBPB* and the unspecified effect of *BATF* on *BCL2L1*) are pruned because in all cases the acting transcription factor is down-regulated. Consequently, these interactions do not have any effect, neither on gene expression nor on network stability.

Table S1.1

|  | Gm12878 | H1-hESC | K-562 | HepG2 |
| --- | --- | --- | --- | --- |
| Transcription Factors used for comparison | BATF  BCL11A  BHLHE40  CEBPB  EBF1  ETS1  IRF4  MEF2C  NFE2  PAX5  PBX3  RUNX3  STAT1  STAT5A  ZEB1 | BCL11A  CEBPB  POU5F1  RXRA | BHLHE40  E2F6  ETS1  GATA2  KAT2B  NFE2  NR2F2  STAT1 | ARID3A  CEBPB  ELF1  FOXA1  HNF4A  NR2F2  RXRA  TCF7L2  TEAD4 |

Table S2: Transcription factors used for comparing the specificity of our derived networks for six examples.

***HepG2/H1-hESC***

The second example studies the phenotypical differences between HepG2 and H1-hESC. Like in the first example, we used cutoffs of 0.001 for the p-value and a fold change greater than 4 and obtained 1049 differentially expressed genes. According to the nature of both phenotypes, the large number of differentially expressed genes is not surprising. However, only 442 of these genes have in total 1043 reported interactions in MetaCore. Like in the previous case we used ChIP-seq data for 9 and 3 TFs for the different phenotypes. Initially, the interaction map contains 122 and 20 interactions reported in ChIP-seq, respectively.

The HepG2 specific network includes 111 out of previously reported 122 interactions. As previously described above, in this case the missing interactions are not relevant in terms of network stability or gene expression matching. The eleven interactions pruned during the contextualization are: activation of *CDH3* by *CEBPB*, the unspecified effects on *LPHN3* and *CNTN1* by *CEBPB*, the inhibitions of *SERPINC1* and *GPAM* by *FOXA1*, the inhibitions of *NFE2L2* and *OSGIN1* by *HNF4A* and the inhibitions of *APOC3*, *APOA2*, *APOA1* and *FABP1* by *NR2F2*. All inhibitory interactions are pruned because of inconsistencies with the gene expression profile of those genes. In all cases the interacting genes are up-regulated and including the inhibitory effects would result in a mismatch. The activation of *CDH3* by *CEBPB* is also not consistent with gene expression, since *CEBPB* is up-regulated whereas *CDH3* is down-regulated. The remaining interactions with unknown biological effect are pruned because they are redundant.

Out of 20 interactions reported for H1-hESC, the inferred network contains 13. The seven missing edges are: The activations of *BCL2L1* and *MFGE8* by *CEBPB*, the activation of *CYP26A1* by *RXRA* and the unspecified interactions of *CEBPB* acting on *GFPT2*, *IDO1*, *SULT2A1* and *CNTN1*. In this network, CEBPB is considered to be up-regulated whereas BCL2L1 and MFGE8 are down-regulated. Consequently, the activation is not consistent with gene expression and hence these edges have to be pruned. Since RXRA is down-regulated it cannot act as an activator for CYP26A1 and hence the pruning is consistent and just removes redundancies in the network. For the other four interactions with unknown sign the gene expression is consistent due to the interactions with other genes in the networks.

***HepG2/K-562***

In this example, we used HepG2 and K-562 to derive a list of differentially expressed genes setting a fold-change threshold of 4 and a p-value less than 0.001. We obtained 774 differentially expressed genes of which 303 contained interactions in MetaCore. The initial interaction map contains 606 interactions out of which the effect of 126 interactions is unspecified and thus subject of sign prediction. Out of this 303 genes in the network, we obtained ChIP-seq data for 9 and 8 Transcription Factors from ENCODE for each phenotype, respectively. The initial network contains 74 reported interactions for HepG2 and no interactions for K562. Therefore, we focus in the following on the HepG2 network.

After contextualization, the derived network still contains 71 interactions reported in ChIP-seq. Consequently, we examined the three pruned interactions. The first interaction is the inhibition of *SERPINC1* by *FOXA1*. According to the differential expression, both genes are up-regulated, showing a clear inconsistency between gene expression and the type of interaction reported, further highlighting the correctness of the pruning. The second interaction is the unspecified effect of *HNF4A* on *C2*. Pruning this edge is a result of the procedure for building the consensus network out of the solutions generated by our algorithm, as in principle this interaction is consistent with gene expression data. Since other genes also regulate *C2*, supporting its expression, this interaction is found to be redundant and not necessary in terms of expression or network stability. The last missing interaction is the inhibition of *FN1* by *HNF4A*. Since both genes are considered to be up-regulated, the pruning of this interaction is necessary to preserve the expression value of *FN1*.

***GM12878/H1-hESC***

Like in the example of HepG2 and H1-hESC we employed a p-value of 0.001 and a fold change greater than 4 to derive 1064 differentially expressed genes of which 202 build the basis of our initial interaction map. Out of these 202 genes we obtained ChIP-seq data for 3 TFs in GM12878 and no for H1-hESC. Only two of the reported interactions are represented in our initial network. After contextualization, these two interactions are preserved.

***GM12878/K-562***

For this example, we employed a less strict criterion than in the others. Namely, we constrain our t-test with a p-value of 0.05 and a fold change of 4 and obtained 1239 differentially expressed genes. Almost half of these genes have reported interactions among each other in literature. Then, we compiled data for the phenotype-specific ChIP-Seq networks, including 15 and 8 TFs in each case, respectively.

The GM12878 specific network contains 79 out of 88 interactions reported in ChIP-seq. The missing interactions are: The inhibition of *OAS1* and *PMAIP1* by *IRF4*, the inhibition of *NCF4*, *PRDM1* and *MYCBP2* by *PAX5*, the activation of *PIM1* and *MAP1A* by *STAT1*, the inhibition of *NTRK2* by *RUNX3* and the unspecified effect of *RUNX3* on *ITGA5*. All the inhibitory interactions are pruned because the interacting genes are up-regulated, leading to gene expression inconsistencies. The activations of *PIM1* and *MAP1A* by *STAT1* are pruned because both *PIM1* and *MAP1A* are down-regulated and the unspecified effect of *RUNX3* on *ITGA5*, which could be considered as an inhibition, was pruned after the consensus of the best solutions was obtained.

In case of K-562 the network contains 13 out of 18 interactions. The five pruned interactions are: activation of TYRO3 and MGAT5 by ETS1, the activation of HLA-E by STAT1, and the unspecified effects of E2F6 on ARNTL2 and GATA2 on TNFAIP3. The unknown effect interaction of GATA2 and TNFAIP3 is pruned after the generation of the consensus of the solutions after contextualization. Since GATA2 is up-regulated and TNFAIP3 is down-regulated there are two possibilities for this interaction. Either it is an inhibition or it has to be pruned. In any case GATA2 is the only up-regulated TF acting on TNFAIP3, which in turn allows both possibilities. All the other edges result from a down-regulated transcription factor and thus can fairly be neglected since they do not further contribute to network stability or gene expression explanation.

***H1-hESC/K-562***

Our last example analyzes the differential network between H1-hESC and K-562. Using a p-value of 0.001 and a fold change greater than 4, we obtained 1045 differentially expressed genes. Our initial literature interaction map then consists of 456 of these genes having 1039 interactions among each other. We obtained ChIP-seq data for 4 TFs for H1-hESC and 8 TFs for K-562. In the case of the H1-hESC specific network, the literature interaction map contains 4 interactions reported in ChIP-Seq. All of these four interactions are also present in the contextualized network.

The K-562 specific network, in contrast, contains also 4 interactions out of which three are represented in the phenotype-specific network. The only missing interaction is the activation of *TSC22D1* by *BHLHE40*. Since the expression of both genes is down-regulated in K-562, this interaction does not contribute to gene expression determination. According to the network structure it also does not contribute to network stability. Thus it can be discarded and the pruning has been shown to be valid in this case.

Table S1.2

|  | ***HepG2/GM*** | | ***HepG2/H1*** | | ***HepG2/K562*** | | ***GM/H1*** | | ***GM/K562*** | | ***H1/K562*** | |
| --- | --- | --- | --- | --- | --- | --- | --- | --- | --- | --- | --- | --- |
|  | ***HepG2*** | ***GM*** | ***HepG2*** | ***H1*** | ***HepG2*** | ***K562*** | ***GM*** | ***H1*** | ***GM*** | ***K562*** | ***H1*** | ***K562*** |
| ***Raw*** | ***92*** | ***36*** | ***122*** | ***20*** | ***74*** | ***0*** | ***2*** | ***0*** | ***88*** | ***18*** | ***4*** | ***4*** |
| ***Pruned*** | ***86*** | ***30*** | ***111*** | ***13*** | ***71*** | ***0*** | ***2*** | ***0*** | ***79*** | ***13*** | ***4*** | ***3*** |
| ***Pruning***  ***Ok?*** | ***🗸*** | ***🗸*** | ***🗸*** | ***🗸*** | ***🗸*** | ***🗸*** | ***🗸*** | ***🗸*** | ***🗸*** | ***🗸*** | ***🗸*** | ***🗸*** |

Table S1.2: Results of ChIP-Seq comparison for six examples. The pruning is considered to be ok if all of the missing interactions were due to a) incompatible expression, b) network stablitity or c) redundancy. On average 89% of the interactions reported in ChIP-Seq are retained in the contextualized networks.

Figure S1


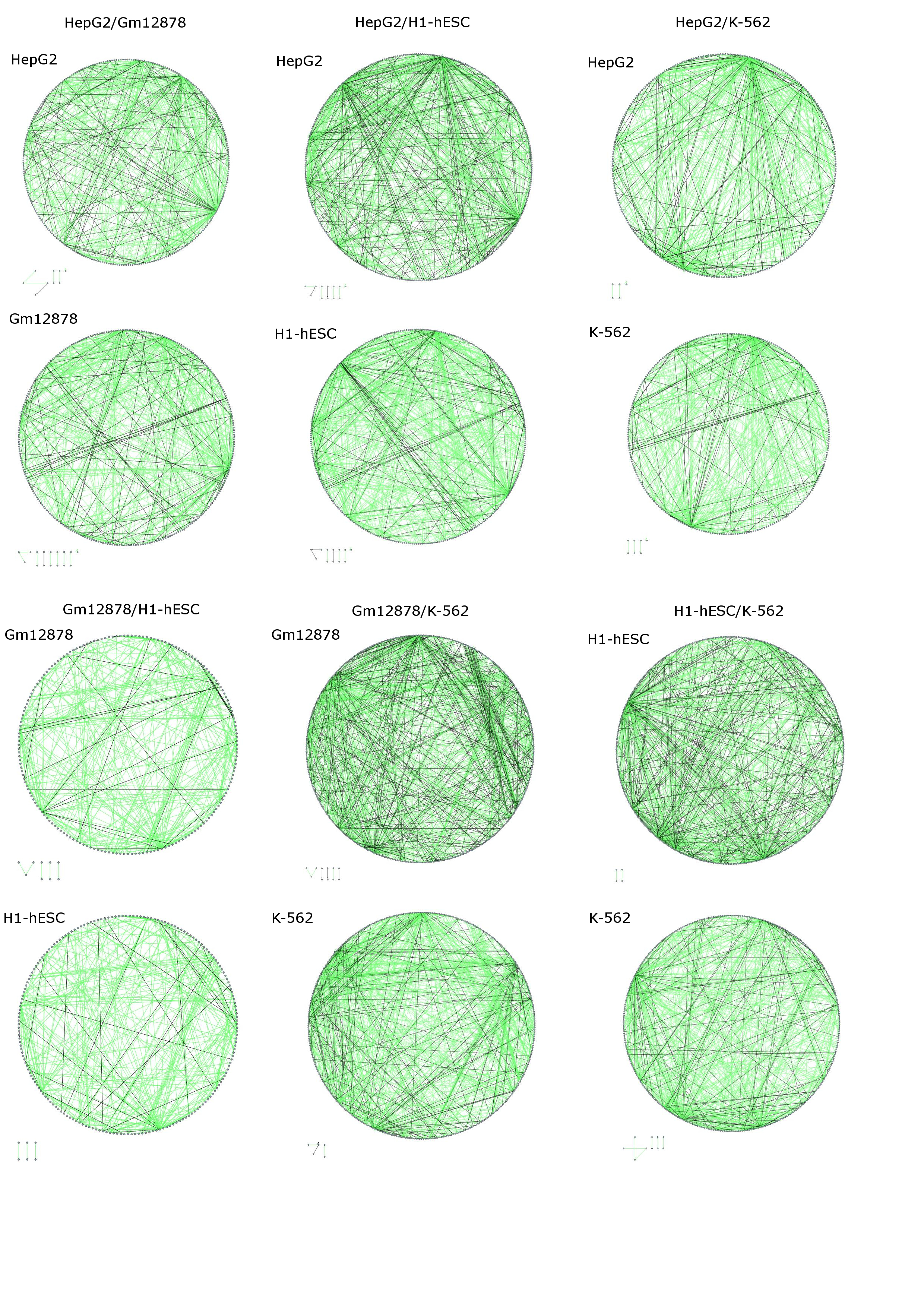


Figure S1: The derived networks for six examples showing the common (green) and phenotype-specific interactions (black). In case of Gm12878/H1-hESC both phenotypes share 92% of the interactions, indicating that a differential network is not needed. However, the network inference algorithm identifies this case and returns similar networks. The number of phenotype-specific interactions increases up to 33.7% in case of Gm12878/K-562. In other cases, the numbers of phenotype-specific interactions range from 14% up to 30.8%. This underscores the need for a differential network approach.
