# Supplementary material for: Prediction of disease–gene–drug relationships following a differential network analysis
Source: Cell Death Dis. 2016 Jan 14;7(1):e2040–. doi: 10.1038/cddis.2015.393 (PMC4816176; doi:10.1038/cddis.2015.393)
Supplement: Supplementary Material S2 [file cddis2015393x2.docx]

Supplementary Material S2

**Comparison of our methodology to other methods available for phenotype-specific network reconstruction**

We compared our method to reconstruct phenotype-specific gene regulatory networks based on initial literature interaction maps against two similar inference methods, SigNetTrainer^1^ and CellNOptR^2^. Similarly to our method, SigNetTrainer and CellNOptR rely on interaction maps encompassing signed –i.e. interactions with a known mechanism of action (activation or inhibition)– and directed gene interactions –i.e. interactions with a known regulator🡪regulated gene directionality. SigNetTrainer determines the optimal sub-graph within the initial interaction map that better describes the expression pattern provided as input to the program, by solving an integer linear programming (ILP) problem. Similarly to our approach, CellNOptR constructs a Boolean network model based on the provided interaction map that better satisfies the supplied expression pattern. However, CellNOptR follows a completely different strategy than the method presented in this paper, as it iteratively adds interactions until the model satisfies the expression pattern or a predefined number of iterations are exceeded. In contrast, our method prunes interactions from the initial interaction maps to converge into the best GRN topology explaining the gene expression data.

We compared our method with CellNOptR and SigNetTrainer based on two criteria, the ability to reconstruct GRNs that explain the given gene expression profile, and the enrichment in experimentally validated interactions (ChIP-Seq interactions from ENCODE) in the reconstructed GRNs. As a benchmarking dataset, we used the initial interaction maps of the six examples used for validating our network inference approach (see Supplementary File S1), encompassing 13 gene interaction maps including experimentally validated interactions, and one core GRN controlling MEP cell fate commitment^3^.

In the comparison with CellNOptR, we used the same parameters for all the interaction datasets analyzed, namely a population size of 1000, 200 generations, a crossover probability of 0.9 and a mutation probability of one interaction per network. For the comparison with SigNetTrainer we build a profile GRN model from the best 100 solutions generated by this method. In general, we followed the parameter setting proposed by the reference manuals except that we increased the population size and decreased the mutation rate for CellNOptR, since we did observed no convergence for the original set of parameters (population size 100 and mutation rate 0.5). Since SigNetTrainer solves an ILP problem, it does not need a specification for the parameters determining the generation size, crossover and mutation probabilities. However, the population size determines the number of GRN configurations out of which a consensus solution is built, as in the case of our method. In the following we will discuss the results of the comparison against CellNOptR and SigNetTrainer.

**Comparison of our method with CellNOptR**

The results for the network reconstruction for all the benchmarking examples are included in Table S2.1. Notably, CellNOptR was not able to process six of the examples included in the benchmarking dataset. In contrast, our method reconstructed the networks in all cases within 12 to 16 minutes of computer system time. The time until the optimization completed is neither linearly dependent on the number of nodes nor on the number of edges. Instead, the population size and network structure –i.e. the number of iterations needed for reaching a steady state– determine the runtime. Of the thirteen subnetworks constructed from the aforementioned examples, CellNOptR was not able to process four. For the reconstruction of the other datasets that were processed with this method, the phenotypic differences observed between the attractor of the reconstructed network and the real expression pattern are below 5 % in most of the cases. Notably, CellNOptR was able to explain only 48% of the phenotype in the HepG2/Gm12878_Sub1 example, whereas with our approach the accuracy is 93%. In the case of HepG2/K562_Sub2 both our approach and CellNOptR could not reach an explanation of 90% of the phenotype. However, the enrichment in ChIP-seq validated interactions in the reconstructed networks obtained with CellNOptR is on average only 58%, which is significantly lower than the 94% of enrichment obtained with our method. In terms of runtime, CellNOptR outperforms our approach and is at least twice as fast in processing the benchmarking datasets. Remarkably, the results obtained for the literature validated core network mediating MEP cell fate commitment^3^ show that CellNOptR only retains 6 out of 27 experimentally validated interactions, whereas our approach is able to retain all of them. In conclusion, our approach is able to generate more accurate reconstructed network models than CellNOptR, as the enrichment in experimentally validated interactions in the different benchmarking datasets analyzed is on average the double with our method in comparison with CellNOptR.

**Comparison of our method with SigNetTrainer**

For comparing the accuracy of SigNetTrainer we used the same set of benchmarking datasets described in the previous section. The results obtained are shown in detail in Table S2.1. Similarly to CellNOptR, SigNetTrainer was not able to reconstruct networks for the six of the benchmarking datasets analyzed. The results in Table S2.1 show that our method outperforms SigNetTrainer for reconstruction of networks compatible with the phenotype-specific gene expression patterns, as the accuracy for the explanation of the gene expression pattern obtained with our approach is similar or higher in all the benchmarking datasets. In particular, in some cases such as Gm12878/H1_Sub and HepG2/K562_Sub2 our method shows an increased accuracy of 27% and 11%, respectively. On average our method shows an accuracy of 94.24% compared to 88.63% of SigNetTrainer. These differences are consequently also reflected in the number of retained ChIP-Seq experimentally validated interactions retained in the reconstructed networks. The reconstructed networks obtained with our method are on average more enriched in ChIP-seq interactions in comparison with SigNetTrainer (97.91% compared to 83.29% achieved with SigNetTrainer). One notable example is HepG2/H1_Sub3, in which the phenotypic accuracy obtained with both methods is identical (94.74%), and the enrichment in ChIP-Seq validated interactions obtained with our method is 50% higher than the one obtained with SigNetTrainer (34 out of 35, and 12 out of 35 respectively). Overall, similarly to the comparison of our method with CellNOptR, our method allows the reconstruction of more accurate networks explaining the phenotype-specific gene expression pattern, with a high enrichment in experimentally validated interactions in comparison with SigNetTrainer.

**Computing environment**

All analyses were conducted on a Mac Pro with 3.7GHz Quad-Core Intel Xeon E5 processor and 16GB RAM.

Table S2.1. Comparison statistics for 21 networks

|  |  |  | Our methodology | | | SigNetTrainer | | | CellNOptR | | |
| --- | --- | --- | --- | --- | --- | --- | --- | --- | --- | --- | --- |
| Interaction Dataset | #Nodes | #Edges | Phenotype | ChIP-Seq | Runtime | Phenotype | ChIP-Seq | Runtime | Phenotype | ChIP-Seq | Runtime |
| Gm12878/H1 | 201 | 356 | 0.8209 | 2/2 | 12 min 38s | --- | --- | --- | --- | --- | --- |
| Gm12878/K562 | 502 | 1275 | 0.8267 | 79/88 | 15 min 9s | --- | --- | --- | --- | --- | --- |
| H1/K562 | 456 | 1039 | 0.9211 | 4/4 | 14 min 32s | --- | --- | --- | --- | --- | --- |
| HepG2/Gm12878 | 344 | 665 | 0.9099 | 86/92 | 16 min 17s | --- | --- | --- | --- | --- | --- |
| HepG2/H1 | 442 | 1043 | 0.8281 | 30/36 | 15 min 55s | --- | --- | --- | --- | --- | --- |
| HepG2/K562 | 303 | 606 | 0.9175 | 111/122 | 13 min 29s | --- | --- | --- | --- | --- | --- |
| Gm12878/H1_Sub | 36 | 61 | 1 | 2/2 | 9 min 37s | 0.7222 | 2/2 | 4 min 5s | 0.9444 | 0/2 | 3 min 10s |
| Gm12878/K562_Sub1 | 38 | 105 | 0.9474 | 37/41 | 10 min 41s | 0.8947 | 31/41 | 44 min 63s | 0.9737 | 26/41 | 5 min 48s |
| Gm12878/K562_Sub2 | 39 | 129 | 0.8974 | 14/15 | 11 min 35s | 0.8974 | 13/15 | 4 min 11s | --- | --- | --- |
| Gm12878/K562_Sub3 | 38 | 118 | 0.9210 | 7/7 | 10 min 41s | 0.8947 | 4/7 | 1h 47 min 26s | --- | --- | --- |
| H1/K562_Sub | 24 | 61 | 0.9583 | 3/3 | 9 min 2s | 0.7916 | 1/3 | 46 min 33s | --- | --- | --- |
| HepG2/Gm12878_Sub1 | 78 | 137 | 0.9358 | 63/67 | 10 min 55s | 0.8589 | 62/67 | 30 min 13s | 0.4872 | 15/67 | 6 min 53s |
| HepG2/Gm12878_Sub2 | 32 | 90 | 0.9688 | 32/33 | 10 min 36s | 0.9375 | 30/33 | 3 min 13s | 0.9999 | 20/33 | 4 min 27s |
| HepG2/H1_Sub1 | 15 | 45 | 0.9333 | 17/19 | 7 min 40s | 0.9333 | 17/19 | 4 min 28s | 0.9999 | 8/19 | 3 min 51s |
| HepG2/H1_Sub2 | 77 | 155 | 0.9481 | 85/94 | 19 min 33s | 0.8961 | 72/94 | 1h 37 min 34s | 0.9999 | 71/94 | 8 min 4s |
| HepG2/H1_Sub3 | 38 | 164 | 0.9474 | 34/35 | 11 min 1s | 0.9474 | 12/35 | 8 min 57s | --- | --- | --- |
| HepG2/K562_Sub1 | 11 | 27 | 1 | 13/14 | 7 min 29s | 1 | 13/14 | 47s | 0.9999 | 7/14 | 2 min 21s |
| HepG2/K562_Sub2 | 26 | 59 | 0.8077 | 3/3 | 8 min 58s | 0.6923 | 3/3 | 12 min 31s | 0.8846 | 2/3 | 3 min 33s |
| HepG2/K562_Sub3 | 70 | 189 | 0.9286 | 38/38 | 12 min 47s | 0.9428 | 37/38 | 26 min 29s | 0.9714 | 32/38 | 8 min 50s |
| MEP Cell Fate | 8 | 27 | 1 | 27/27 | 5 min 13s | 1 | 22/27 | 6 min 16s | 0.9999 | 6/27 | 4 min 3s |

**Legend**

**Table S2.1**

A total of 21 networks were generated to compare our network inference algorithm against CellNOptR and SigNetTrainer. The first six networks are the result of a differential expression test (for Details see Supplementary File S1), while all networks containing “Sub” are sub-networks extracted from the complete networks described above. We also included the core network mediating MEP cell fate commitment obtained from literature^3^. We include information of the number of nodes and edges in each network, as well as the phenotypic agreement of the network attractor with the real expression pattern, the enrichment of ChIP-Seq validated interactions and the runtime. The “Phenotype” column gives information about the overlap of the network attractor and the experimentally validated steady state. A value of 1 corresponds to 100% overlap whereas a value of 0 indicates no overlap. The “ChIP-Seq” column provides the number of ChIP-Seq validated interactions retained in the reconstructed networks in comparison to the number of validated interactions in the initial interaction maps. Fields containing “---“ indicate that the corresponding method was not able to converge into a solution.

Bibliography

1 Melas IN, Samaga R, Alexopoulos LG, Klamt S. Detecting and removing inconsistencies between experimental data and signaling network topologies using integer linear programming on interaction graphs. *PLoS Comput Biol* 2013; **9**: e1003204.

2 Terfve C, Cokelaer T, Henriques D, MacNamara A, Goncalves E, Morris MK *et al.* CellNOptR: a flexible toolkit to train protein signaling networks to data using multiple logic formalisms. *BMC Syst Biol* 2012; **6**: 133.

3 Doré LC, Crispino JD. Transcription factor networks in erythroid cell and megakaryocyte development. *Blood* 2011; **118**: 231–9.
