# Supplementary material for: Prediction of disease–gene–drug relationships following a differential network analysis
Source: Cell Death Dis. 2016 Jan 14;7(1):e2040–. doi: 10.1038/cddis.2015.393 (PMC4816176; doi:10.1038/cddis.2015.393)
Supplement: Supplementary Material S3 [file cddis2015393x3.docx]

Supplementary Material S3

**Interaction sign inference analysis**

The network inference approach presented in this paper infers the mode of action, in the following referred to as the sign of an interaction, that is consistent with the observed expression patterns of the two phenotypes under consideration. However, a consistent assignment is not necessarily in agreement with the actual mode of action. We thus estimated the accuracy of our approach for inferring the sign of interactions. Our analysis is based on a diverse set of networks ranging from 24 to 151 interactions. Since we are not able to test all possible combinations, we tested the performance of our approach to infer the sign of a single interaction. The percentage of correctly inferred signs is used as the base of an exponential distribution. The resulting distribution is then compared against a random sign assignment, obtained by assigning a probability 0.5 for either activation or inhibition, using a two-sample Kolmogorov-Smirnov test (KS-test), with significance level 0.1. We tested the null hypothesis that both observations originate from the same distribution against the alternative hypothesis that our obtained observations tend to be higher than the ones from a random distribution. If the ratio of correctly assigned interactions obtained with our method is higher than those obtained using a random assignment, the empirical CDF of our method should be smaller than the one obtained from a random assignment. The results of the KS-Tests are shown in Table S3.1. In general, the results obtained with our approach are significantly higher than those a obtained following a random assignment. In all but one case, the KS-test rejects the null hypothesis (KS-test result is 1), for a significance level of 0.1. However, in one case there is no clear distinction between the sign assignment obtained using our method and random assignment. This can be explained by the small number of interactions in this network, which hampers the possibility of detecting a significant difference. Nevertheless, we observe in all cases an increased percentage of correctly predicted interaction signs.

Table S3.1. Mode of action inference statistics

|  | #Edges | Single sign restored | KS-Test decision | p-value |
| --- | --- | --- | --- | --- |
| Network 1 | 42 | 0.69047619 | 1 | 3.9795e-05 |
| Network 2 | 48 | 0.604166667 | 1 | 0.0132 |
| Network 3 | 74 | 0.662162162 | 1 | 2.9889e-06 |
| Network 4 | 152 | 0.6 | 1 | 1.9424e-05 |
| Network 5 | 25 | 0.56 | 0 | 0.3371 |
| Network 6 | 48 | 0.583333333 | 1 | 0.0693 |

Legend

Table S3.1

The table shows the networks generated for assessing the mode of action inference. The percentage of correctly identified modes of action upon removal of a single sign is shown. Our sign inference approach is performing in all but one case statistically better than a random assignment. In case of Network 5, the number of edges is too small in order to detect statistical significance. A KS-Test decision of 1 indicates that the null hypothesis that both distributions are the same is rejected, and 0 indicates that the null hypothesis is accepted.
