# Supplementary material for: Prediction of disease–gene–drug relationships following a differential network analysis
Source: Cell Death Dis. 2016 Jan 14;7(1):e2040–. doi: 10.1038/cddis.2015.393 (PMC4816176; doi:10.1038/cddis.2015.393)
Supplement: Supplementary File S4 [file cddis2015393x4.docx]

**Supplementary File S4**

**Parameter statistics for network inference**

In this section we assess for which parameters our network inference algorithm provides reliable results. We used the same 13 sub-networks as for the comparison to other methods (see Supplementary File S2) and inferred the networks for 3025 different parameter sets. In particular, we defined the parameter ranges as shown in Table S4.1. For each possible set of parameters, we computed the mean and variance of the resulting fitness distribution over all individuals after the specified number of generations. Next, we clustered the quadruples containing the four moments using k-means clustering. In order to determine the optimal number of clusters, we determined the silhouette value for each point containing the mean and variance, defined as $S_{i}=\frac{b_{i}-a_{i}}{max(a_{i},b_{i})}$. Here, $a_{i}$ is the average distance from the i-th point to the other points in the same cluster as *i,* and $b_{i}$ is the minimum average distance from the i-th point to points in a different cluster. The silhouette value ranges from -1 to +1 where higher values indicate a better match to its own cluster. We thus determined the number of clusters maximizing the sum of silhouette values.

Table S4.1. Parameter ranges

|  | Min value | Max value | Step Size |
| --- | --- | --- | --- |
| Population | #edges | 5*#edges | #edges |
| Generations | 100 | 500 | 100 |
| Mutation rate | 0.0 | 0.5 | 0.05 |
| Crossover rate | 0.0 | 1.0 | 0.05 |

With the resulting cluster containing the best solutions, we performed a frequency analysis of the parameters (see Figure S4.1). It is evident, that the population size and the crossover rate (Figure S4.1A and D) are influencing the optimal solutions less. On the other hand, the number of solutions is highly dependent on the number of generations and the mutation rate (Figure S4.1B and C). The number of generations positively correlates with the number of solutions in the best cluster indicating the preference of more iterations over a bigger population size. On the other hand, our results indicate that the mutation rate has to be chosen between 0 and 0.05 in order to obtain optimal solutions. The obtained solutions coincide with our choice of the parameters for which the results of this paper were obtained.

Figure S4.1. Parameter statistics


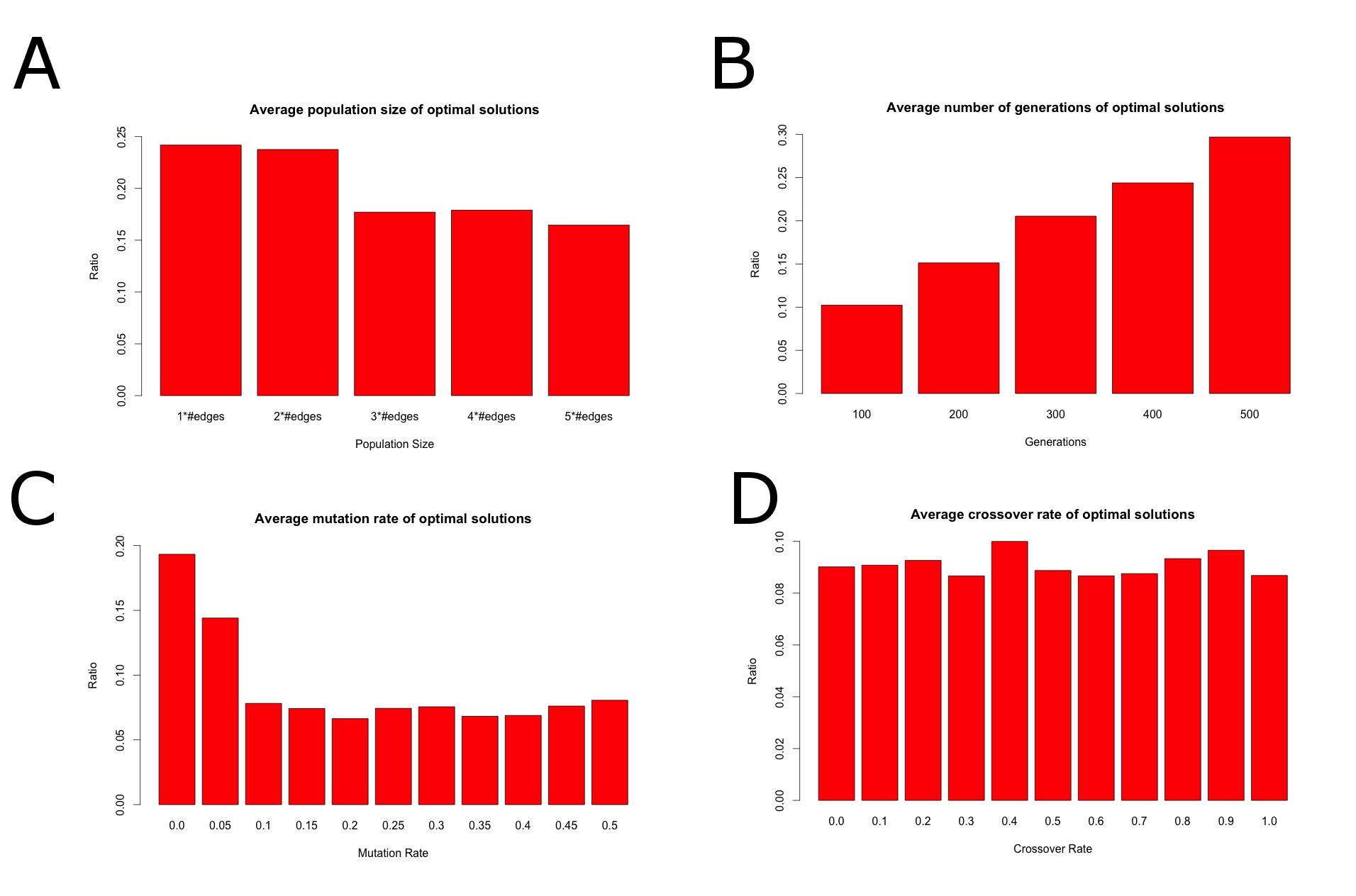


Legend

Table S4.1.

The parameter ranges included in the statistics are given. The mutation rate is cut off at a maximum value since the algorithm tends to not converge anymore. The number of unknowns in the network determined the minimum value of the population size. The ranges were discretized by setting a step value according to the “Step Size” column.

Figure S4.1.

The percentage of top ranking solutions obtained for different parameters. (A) It is sufficient to take up to twice as many individuals than there are unknowns (edges) in the network. (B) A linear correlation of the probability to obtain a top ranking solution and the number of generations has been found. For the parameters tested 500 generations was the best value. (C) The mutation rate has to be chosen between 0 and 0.05 for the highest chance of obtaining a top ranking solution. (D) For the crossover rate no clear trend can be observed. The choice of this parameter can be neglected.
